# Susceptibility to primary angle closure glaucoma in Saudi Arabia: the possible role of mitochondrial DNA ancestry informative haplogroups

**Published:** 2011-08-12

**Authors:** Khaled K. Abu-Amero, Ana M. González, Essam A. Osman, José M. Larruga, Vicente M. Cabrera, Saleh A. Al-Obeidan

**Affiliations:** 1 Department of Ophthalmology, College of Medicine, King Saud University, Riyadh, Saudi Arabia; 2 Department of Genetics, Faculty of Biology, University of La Laguna, Tenerife, Spain; 3 Department of Ophthalmology, College of Medicine, University of Florida, Jacksonville, FL

## Abstract

**Purpose:**

In a previous preliminary analysis we reported that mitochondrial DNA (mtDNA) haplogroup R0a was significantly more frequent in primary angle closure glaucoma (PACG) Saudi patients than in healthy Saudi controls. This result prompted us to extend our work using a significant larger Saudi PACG cohort and more healthy controls.

**Methods:**

We sequenced the mtDNA regulatory hypervariable region-I (HVS-I) and coding regions, comprising haplogroup diagnostic polymorphisms, in 227 PACG Saudi patients and compared their haplogroup frequencies with those obtained from 186 matched healthy controls (free of PACG by examination) and from a large sample of 810 healthy Saudi Arabs representing the general Saudi population.

**Results:**

MtDNA Haplogroups R0a and J, the most abundant lineages in Saudi Arabia, were in significant higher frequencies in the PACG patients than in controls, while the widespread western Eurasian haplogroup U was associated with reduced risk to developing PACG.

**Conclusions:**

Haplogroups R0a and J could be ancestry informative markers for PACG in the Saudi Arabian population. In addition, the western Eurasian haplogroup U may play a mild protective effect to this illness.

## Introduction

Although glaucoma embodies a heterogeneous group of optic neuropathies, all types are defined by progressive and irreversible degeneration of the optic nerve with gradual visual field loss. Within this group, primary angle-closure glaucoma (PACG) is a type of glaucoma characterized by a narrow iridocorneal angle resulting in a blockage of the aqueous outflow structures. It seems that there is an anatomic and physiologic predisposition to PACG. Thus, shallow anterior chamber depth is considered an anatomically inheritable risk factor for this illness [1]. Congruently, Eastern Asian ascendance, hyperopia and female gender significantly predispose to this disease [2]. It has been estimated that in Saudi Arabia, 40% of glaucoma patients belong to the PACG type [3]. This percentage is closer to those calculated for Asian than for European populations [4]. Although a hereditary component for PACG exists, causative genes have not yet been identified and only occasional differences in gene frequency polymorphisms or in levels of gene expression between PACG patients and healthy controls have been reported. For instance, mutations in the myocilin (*MYOC*) gene were observed in PACG Chinese patients but also in Chinese controls ruling out a role of this gene in the pathogenesis of PACG [5]. The C677T polymorphism in the methylenetetrahydrofolate reductase (*MTHFR*) gene was found associated with PACG in a Pakistani cohort [6], but this result has not been extended to other populations. The possible role of mitochondrial abnormalities (mtDNA mutations and respiratory activity) in PACG was investigated and authors found minimal evidence of such abnormalities [7]. A significant increase of SPARC (secreted protein, acidic, and rich in cysteine) in the iris of PACG patients compared to healthy controls has also been documented pointing to a possible role of this gene in the development of PACG [8]. However, these results have not yet been confirmed. PACG is also characterized by an increase of intraocular pressure (IOP). Recently it has been demonstrated experimentally that elevated hydrostatic pressure induces mitochondrial structural changes and apoptotic cell death which suggests, once more, a possible role of mitochondrial injury in glaucoma [9]. Significant associations between mitochondrial DNA (mtDNA) haplogroups and different types of glaucoma have been detected in the Saudi Arabia population [3,10]. Furthermore, in a preliminary pilot experiment, a highly significant prevalence of mtDNA haplogroup preHV1 (now named R0a) in a small cohort of 29 PACG Saudi patients was found [11]. The aim of the present study was to evaluate the possible role of mtDNA Haplogroup(s) in large cohort of PACG Saudi patients with special emphasis to confirm the susceptibility to PACG for carriers of R0a, the most representative mtDNA haplogroup in the Arabian peninsula [12].

## Methods

### Patients and control subjects

We recruited 227 Saudi PACG patients who satisfied strict clinical criteria for PACG. Patients were eligible for inclusion if they had evidence of glaucomatous optic nerve damage attributable to primary angle closure [13]. Inclusion criteria included at least three of the following: 1) clinical documentation of angle closure, defined as the presence of appositional or synnechial closure of the anterior chamber angle involving at least 270 degrees by gonioscopy in either eye; 2) intraocular pressure elevated to a level ≥21 mmHg measured by Goldmann applanation tonometry before or after treatment; 3) evidence of characteristic glaucomatous optic disk damage with excavation of the disc causing a cup-to-disk ratio (c/d) vertically of at least 0.70 in at least one eye; and 4) characteristic peripheral visual field loss including nerve fiber bundle defects (nasal step, arcuate scotoma, paracentral scotoma) or advanced visual field loss (central and/or temporal island of vision) as tested by Humphrey Field Analyzer in those patients with vision better than 20/200 or Goldmann Manual Perimetry in those with worse vision. Exclusion criteria included: 1) secondary angle closure glaucoma; 2) presence of pseudoexfoliation syndrome even if coexistent with angle closure; 3) another cause of optic nerve injury affecting either eye; 4) significant visual loss in both eyes not associated with glaucoma; 5) inability to visualize the fundus for optic disk assessment; or 6) refusal to participate. Patients were recruited from the glaucoma clinic at King Abdulaziz University Hospital (KAUH) after signing an informed consent approved by the institutional review board (proposal number # 08–657).

A second group (n=186) of healthy Saudi Arabs controls (HMC group) free from glaucoma by examination were recruited. Entry criteria for those subjects were age >40, normal IOP, open angles on gonioscopy, and normal optic nerves on examination.

A third large sample (n=810) of healthy Saudi Arabs (HSA), representing all five major Saudi Arabian provinces, that were recruited previously for population genetics studies, was used for statistical comparisons. All individuals of this group were Saudi Arabs who reported no symptomatic metabolic, genetic, ocular disorders or any ophthalmic problem on an extensive questionnaire regarding family history, past medical problems, and current health. For those controls, their mother’s ancestral origin was established as a Saudi. The mtDNA haplogroup assignation for 552 subjects of this sample has been already published [11]. All patients and controls were maternally unrelated Saudi Arabs, all whose known ancestors were of Saudi Arabian origin. This research followed the tenets of the Declaration of Helsinki.

### DNA extraction

Five milliters of peripheral blood were collected in EDTA tubes from all participating individuals. DNA was extracted using the illustra blood genomicPrep Mini Spin Kit from GE Healthcare (Buckinhamshire, UK), and stored at −20 °C in aliquots until required.

### Mitochondrial haplogroup assortment

All samples were amplified and sequenced for the mtDNA regulatory region hypervariable segment I (HVS I) I using previously described primers and conditions [14] (Table 1). Briefly, each 25 µl PCR reaction contained 2.5 µl of 10× reaction buffer with MgCl_2_ (Amersham Pharmacia Biotech, Piscataway, NJ), 10 ρmol of each primer, 100 ρmol/µl each of deoxynucleoside triphophates (deoxyadenosine triphosphate, deoxyguanosine triphosphate, deoxycytidine triphosphate and deoxythymidine triphosphate; Perkin-Elmer Corporation, Foster City, CA) in Tris HCl buffer, 1 unit Taq DNA polymerase (Amersham Pharmacia Biotech, Piscataway, NJ) and 50 ng genomic DNA template. The mixture was denatured at 95 °C for 5 min and the PCR reaction was carried out for 35 cycles, in a GeneAmp 9700 PCR system (Perkin-Elmer Corporation), under the following conditions: denaturation at 95 °C for 1 min, annealing at 54 °C for 45 s, extension at 72 °C for 1 min, and final extension cycle of 72 °C for 7 min. The PCR products were electrophoresed on a 1% agarose gel and detected with 0.5 µg/ml ethidium bromide to confirm the correct amplicon size. Haplotypes were tentatively assorted into haplogroups according to their HVS diagnostic positions. This assortment was further confirmed, when necessary, by the analysis of coding-region diagnostic haplogroup polymorphisms (Table 2), following the most recent mtDNA haplogroup nomenclature [15]. To detect these polymorphisms a fragment spanning the diagnostic position was amplified and sequenced using any of the 32 overlapping pairs of primers that cover the whole mtDNA genome, and the PCR and sequencing conditions previously published for each of them [14].

**Table 1:** Primers used for Mitochondrial Haplogrouping.

Primer #	Primer Sequence	Annealing temp.	Product size
MIT-1-F	CCTCCCTGTACGAAAGGACA	57	614
MIT-1-R	TGAGATTGTTTGGGCTACTGC		
MIT-2-F	CGAGCAGTAGCCCAAACAAT	57	664
MIT-2-R	TTTTGGATTCTCAGGGATGG		
MIT-3-F	CCATCCCTGAGAATCCAAAA	57	653
MIT-3-R	ATTTTGCGTAGCTGGGTCTG		
MIT-4-F	TAAACCAGACCCAGCTACGC	55	661
MIT-4-R	AAAGTGGCTGATTTGCGTTC		
MIT-5-F	TCAACTGAACGCAAATCAGC	60	784
MIT-5-R	TGAAATTGATGGCCCCTAAG		
MIT-6-F	CTTAGGGGCCATCAATTTCA	57	705
MIT-6-R	AAGCCTCCTATGATGGCAAA		
MIT-7-F	GCCATCATAGGAGGCTTCATT	59	702
MIT-7-R	TTTCCTGAGCGTCTGAGATGT		
MIT-8-F	CATCTCAGACGCTCAGGAAA	55	730
MIT-8-R	GGGAGGTAGGTGGTAGTTTGTG		
MIT-9-F	TACTACCGTATGGCCCACCA	60	781
MIT-9-R	AGGCTTGGATTAAGGCGACA		
MIT-10-F	ACTGACTATCCTAGAAATCGCTGT	59	741
MIT-10-R	GCAGATAGTGAGGAAAGTTGAGC		
MIT-11-F	CCACGGACTTCACGTCATTA	59	743
MIT-11-R	GGGAGGATATGAGGTGTGAGC		
MIT-12-F	GCATTTACCATCTCACTTCTAGG	57	751
MIT-12-R	AGTGCGATGAGTAGGGGAAG		
MIT-13-F	AGGCACATACTTCCTATTCTACACC	55	740
MIT-13-R	TGATATTTGATCAGGAGAACGTG		
MIT-14-F	ACTGGGAGAACTCTCTGTGCT	55	740
MIT-14-R	ACGAACAATGCTACAGGGATG		
MIT-15-F	TCCATAATATTCATCCCTGTAGCA	57	741
MIT-15-R	GTGGGTACAGATGTGCAGGA		
MIT-16-F	GTTACAATCGGCATCAACCA	55	740
MIT-16-R	AGCTTTTCTAGTCAGGTTAGGTCTA		
MIT-17-F	CCTCAACCCAAAAAGGCATA	59	745
MIT-17-R	GGAGGTCGATGAATGAGTGG		
MIT-18-F	ACGCAAAATTAACCCCCTAA	59	742
MIT-18-R	GCCTAGGAGGTCTGGTGAGA		
MIT-19-F	CCTAGGCGACCCAGACAAT	59	740
MIT-19-R	GATAGTTGAGGGTTGATTGCTGTA		
MIT-20-F	TGCTTACAAGCAAGTACAGCAAT	57	370
MIT-20-R	TGATGTCTTATTTAAGGGGAACG		
MIT-21-F	GGCTCACATCACCCCATAAA	55	720
MIT-21-R	CATGGGCTACACCTTGACCT		
MIT-22-F	GCAAACCCTGATGAAGGCTA	57	632
MIT-22-R	GGGGTCTTAGCTTTGGCTCT		
MIT-23-F	ACTTTGCAAGGAGAGCCAAA	59	672
MIT-23-R	AGGCGGTGCCTCTAATACTG		
MIT-24-F	TCACCTCTAGCATCACCAGTATT	57	660
MIT-24-R	GGAAGGCGCTTTGTGAAGTA		
HVS-I-F	ACT TCA CAA CAA TCC TAA TCC T	60	1200
HVS-I-R	CGG AGC GAG GAG AGT AGC AC		
HVS-II-F	CAT TTA CCG TAC ATA GCA CA	60	820
HVS-II-R	GGG AGG GGG TGA TCT AAA AC		

**Table 2:** mtDNA haplogroup distribution in PACG patients and controls.

**Haplogroup**	**Coding positions**	**PACG (n=227)**	**HMS (n=186)**	**HSA (n=810)**	PACG * HMS p value	PACG * HSA p value
H	C7028C	14 (6.2%)	9 (4.8%)	66 (8.1%)	0.56	0.32
R0a	T3847C	51 (22.5%)	26 (14.0%)	141 (17.4%)	0.03*	0.08
J	T4216C, C15452A	64 (28.2%)	47 (25.3%)	171 (21.1%)	0.51	0.02*
T	T4216C, G15928A	11 (4.8%)	9 (4.8%)	53 (6.5%)	0.99	0.35
K	A3480G	8 (3.5%)	4 (2.2%)	27 (3.3%)	0.41	0.89
U	A12308G, G12372A	14 (6.2%)	25 (13.4%)	91 (11.2%)	0.01*	0.02*
Other R	C12705T	6 (2.6%)	8 (4.3%)	25 (3.1%)	0.35	0.73
N1	T10238C, G12501A	12 (5.3%)	13 (7.0%)	66 (8.1%)	0.47	0.15
W	G15884C	1 (0.4%)	0	6 (0.7%)	0.37	0.63
X	C6371T	8 (3.5%)	4 (2.2%)	20 (2.5%)	0.41	0.39
M1	C10400T C12403T	6 (2.6%)	11 (5.9%)	25 (3.1%)	0.10	0.73
M(xM1)^1^	C10400T	4 (1.8%)	6 (3.2%)	24 (3.0%)	0.34	0.32
L	T9540C, C10400C	28 (12.3%)	24 (12.9%)	95 (11.7%)	0.86	0.80
L2	T10115C	16 (7.0%)	10 (5.4%)	35 (4.3%)	0.49	0.09
L(xL2)^2^	T9540C, C10400C	12 (5.3%)	14 (7.5%)	60 (7.4%)	0.35	0.27

ACG, Angle-closed glaucoma patients; HMC, healthy matched controls; HAS, healthy Saudi Arab sample. χ^2^ or Fisher exact test were applied to investigate the association between having a certain haplogroup and the occurrence of ACG. **^1^**All M haplogroups except M1. **^2^**All L haplogroups except L2.

### Statistical analysis

Differences in haplogroup frequency between patient and control groups were tested by 2×2 contingence tests or by Fisher's exact tests when appropriate. To keep statistically adequate haplogroup sub-sample sizes within cohorts, when necessary, mtDNA haplogroups were collapsed into larger haplogroup identities following phylogenetic criteria [15]. Genetic diversity, h, within haplogroups and its variance were estimated from the unbiased formulas given by Nei [16], as implemented in the Arlequin 3.5 package. Differences in h were performed using a standard two-tailed z test. Finally the geographic origin distributions of patients and matched controls and differences in haplogroup frequencies among Saudi Arabian provinces were compared by 2×n contingency tests.

## Results

As women have been reported to be at higher risk for PACG than men [17], and as gender was not a selective criteria in the recruitment of patients and controls, we compared the sex-ratio between both cohorts. Among patients, the female to male ratio (1.34:1) was significantly higher (p=0.047) than in controls (0.94), pointing to a greater female susceptibility to PACG in the Saudi population. However, mtDNA haplogroup frequencies did not differ between sexes in any group. Haplogroup frequencies obtained for the PACG patients, healthy matched controls (HMC) and the general Saudi population (HSA) is listed in Table 2. Although mtDNA haplogroup R0a was abundant in PACG (22.5%) than in HMS (14.0%) and HSA (17.4%), only the PACG*HMS comparison reached statistical significance (Table 2). Additionally, Haplogroup J, which is the most representative of the whole Saudi Arabia population [12], was found in higher frequencies in the patients group (28.2%) than in HMS (25.3%) and HSA (21.1%) controls. In this case only the PACG*HSA comparison reached statistical significance (p=0.02). Finally, haplogroup U was significantly underrepresented in PACG patients (6.2%) compared to both the HMS (13.4%) and HSA (11.2%) control groups (Table 2) pointing to a consistent protective effect of this haplogroup to PACG.

## Discussion

By studying the prevalence of various mtDNA hpalogroups in 227 PACG Saudi patients and compare this to a large cohort of population controls (n=810) and to a group of individuals with no PACG by examination (healthy controls, n=186), we found that Haplogroups R0a and J could be ancestry informative markers for PACG in the Saudi Arabian population. In our previous study [11], we also found an association (p=0.00002) between mtDNA haplogroup R0a and PACG in a smaller cohort of Saudi Patients (n=29). The level of significance, for the R0a, found here (p=0.03) is lower than in our previous study [11]. The difference observed in the level of statistical significance could be explained, at first sight, by an ascertainment bias for the previous PACG sampling due to its small sample size (n=29). An associated consequence we could expect of it would be a concomitant diminution in its haplotype diversity (h) within haplogroup R0a. However, h values (Table 3) for the past and present PACG samples are very similar and not significantly different from controls (p=0.38). In addition, our PACG sample seems to be as diverse as controls for the J (p=0.20) and U (p=0.76) for which significant differences between the patient and one or both control cohorts were found (Table 2). Population genetic substructure in Saudi Arabia could also be a possible confounding factor. In fact, it was found in previous population studies that the mtDNA haplogroup frequency distribution in Arabia was geographically structured [12]. Focusing into the haplogroups that showed associations with PACG, there are significant differences in their geographic frequency distributions, across the five main provinces of Saudi Arabia, for R0a (p=0.04), with a frequency range from western (8%) to center (22%), and for J (p=0.002), with a frequency range from western (37%) to northern (16%). Only haplogroup U seems to have a uniform distribution across the country (p=0.63). However, the geographic origin distribution of the recruited PACG patients and HMS matched controls (Table 4) was strikingly similar (p=0.42) discarding wide inter-regional genetic differentiation as a main cause of the PACG and mtDNA haplogroup associations found. With the information at hand, we would attribute the R0a frequency differences found between the two independent PACG samples to biased sampling of the previous one due to its small sample size. However, other demographic population parameters, not yet uncovered, as fine-scale genetic structure and/or hidden social organization should not be discarded.

**Table 3:** Haplotypic (h) diversities within haplogroups in ACG patients and controls.

**Haplogroup**	**R0a**			**J**			**U**		
Cohorts	N	HT	h	N	HT	h	N	HT	h
PACG^1^	15	10	0.84±0.04	3	2	NT	0	0	NT
ACG	51	14	0.88±0.02	64	15	0.76±0.05	14	13	0.99±0.03
HMS	27	13	0.87±0.05	47	18	0.89±0.03	25	18	0.97±0.02
HSA	140	45	0.95±0.01	171	56	0.94±0.01	91	64	0.99±0.04

^1^=previously analyzed [11]. n=haplogroup sample size; HT=Haplotype number; h=haplotype diversity. NT=not tested.

**Table 4:** Geographic origin distribution of ACG patients and controls.

**Region**	**ACG**	**HMS**
Central	102 (0.45)	75 (0.40)
Southern	59 (0.26)	41 (0.22)
Northern	30 (0.13)	36 (0.20)
Western	18 (0.08)	17 (0.09)
Eastern	18 (0.08)	17 (0.09)
Total	227	186

In conclusion, we have detected a significant susceptibility of Saudi R0a carriers to PACG although at a milder level than the one previously reported. It also seems likely that haplogroup J, abundanant among the Saudi population, also confers a mild susceptibility to PACG. On the contrary, the western Eurasian widespread haplogroup U seems to play a mild protective effect to PACG. In a recent analysis on primary open-angle glaucoma (POAG) in Saudi Arabia [3], it was found that mtDNA lineages of sub-Saharan Africa origin seemed to confer susceptibility to POAG, pointing to a role of ancestry informative marker for macro-haplogroup L, as it is well documented that the prevalence of POAG is higher in subjects of African ascendance [18]. In a similar vein, haplogroups R0a and J could reflect a predisposition of the genuine Saudi Arabian population to suffer PACG, which is supported by the high prevalence of this illness in this Country, near of the range observed for South and East Asians, and significantly higher than the one observed for populations of European ascendance [3].
